# Supplementary material for: Electrical tuning of branched flow of light
Source: Nat Commun. 2024 Jan 3;15:197. doi: 10.1038/s41467-023-44500-8 (PMC10764866; doi:10.1038/s41467-023-44500-8)
Supplement: Supplementary file 1 — Supplementary Information [file 41467_2023_44500_MOESM1_ESM.pdf]

Supplementary Information, Chang *et al.*

Supplementary Notes

The supplementary notes are organized as follows. In Supplementary Note 1, the theoretical model of the branched flow of light in a nematic liquid crystal (NLC) medium is discussed. It includes the effective refractive index theory of ordinary wave and extraordinary wave in liquid crystals, the equivalent disordered optical potential of forming a branched flow of light, and the numerical simulations. The scintillation index calculations and dielectric loss effect are also included. In Supplementary Note 2, we introduce the experimental setup and characterizations of the branched flow of light. In Supplementary Note 3, the electrical-tunable and polarization-tunable branched flow in NLC film are discussed. It also includes discussions of tunable potential strength, first branch position, and scintillation index. The simulation of NLC director orientation with electric field is also discussed. The Fréedericksz transition and the temperature tuning method are also discussed. In Supplementary Note 4, the statistical features of branch density are discussed.

Supplementary Note 1: Theoretical model of branched flow of light in nematic liquid crystal

We consider a monochromatic plane wave passing through the nematic liquid crystal (NLC) film, as shown in Fig. S1. The NLC is confined between two glass plates parallel to the *x-y* plane. The NLC orientation field is described by the unit vector (director) $\hat{\mathbf{n}}(\mathbf{r})$. This director describes the average orientation of the symmetry (optical) axis of NLC molecules at position $\mathbf{r}(x, y)$. The director $\hat{\mathbf{n}}(\mathbf{r})$ of NLC molecules is defined by azimuth angle φ and polar angle θ:

$$\hat{\mathbf{n}}(\mathbf{r}) = \begin{bmatrix} \sin\theta\cos\varphi \\ \sin\theta\sin\varphi \\ \cos\theta \end{bmatrix} \quad (1)$$

Assuming that the LC layer is non-magnetic, $\mu = \mu_0$, the permittivity of dielectric anisotropy can be represented by a tensor:

$$\overleftrightarrow{\varepsilon} = \varepsilon_\perp \overleftrightarrow{I} + \Delta\, \varepsilon \hat{\mathbf{n}}\hat{\mathbf{n}} \quad (2)$$

where $\Delta\varepsilon = \varepsilon_\parallel - \varepsilon_\perp$. The extraordinary dielectric constant $\varepsilon_\parallel$ is parallel to the director $\hat{\mathbf{n}}$, and the ordinary dielectric constant $\varepsilon_\perp$ is in the direction perpendicular to the director $\hat{\mathbf{n}}$. In the experiment, we use the normal nematic liquid crystal (NLC), i.e., E7, and $\varepsilon_\parallel > \varepsilon_\perp$.

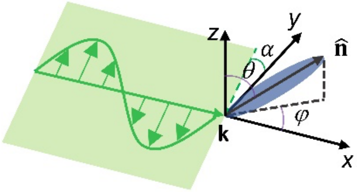

FIG. S1. Schematic of input light on the orientated NLC molecules. The wavevector $\mathbf{k}$ of light is along the x-direction and the linear polarization angle relating to the y-direction in the y-z plane is α. The director $\hat{\mathbf{n}}$ of NLC molecules, is defined by azimuth angle φ and polar angle θ.

The propagation of light in the homogeneous anisotropic NLC medium satisfies Maxwell's equation:

$$\begin{aligned} \nabla \times \mathbf{H} &= -i\omega\overleftrightarrow{\varepsilon}\cdot\mathbf{E} \\ \nabla \times \mathbf{E} &= i\omega\mu_0\mathbf{H} \\ \nabla\cdot(\overleftrightarrow{\varepsilon}\cdot\mathbf{E}) &= 0 \\ \nabla\cdot\mathbf{H} &= 0 \end{aligned} \tag{3}$$

Considering that the monochromatic light with frequency ω is incident along the x-direction, we write $\mathbf{E} = \mathbf{E_0}e^{ikx}$ and $\mathbf{H} = \mathbf{H_0}e^{ikx}$, and wavevector $\mathbf{k} = (k, 0,0)^T$. Combined with Maxwell's equations (3), we get the following relations:

$$-\omega^2\mu_0\overleftrightarrow{\varepsilon}\cdot\mathbf{E_0} = \mathbf{k}\times(\mathbf{k}\times\mathbf{E_0}) \tag{4}$$

After some algebraic calculus, the equations (4) can be further reduced to:

$$(k^2\overleftrightarrow{I} - \mathbf{kk} - \omega^2\mu_0\overleftrightarrow{\varepsilon})\cdot\mathbf{E_0} = 0 \tag{5}$$

The k can be determined by setting $k^2\overleftrightarrow{I} - \mathbf{kk} - \omega^2\mu_0\overleftrightarrow{\varepsilon}$ equal to zero, and we obtain

$$\varepsilon_{xx}k^4 - k^2\omega^2\mu_0\left[(\varepsilon_{yy}+\varepsilon_{zz})\varepsilon_{xx} - \varepsilon_{xz}^2 - \varepsilon_{xy}^2\right] + \omega^4\mu_0^2\left[(\varepsilon_{xx}\varepsilon_{yy} - \varepsilon_{xy}^2)\varepsilon_{zz} - \varepsilon_{xz}^2\varepsilon_{yy} - \varepsilon_{yz}^2\varepsilon_{xx} + 2\varepsilon_{xy}\varepsilon_{yz}\varepsilon_{xz}\right] = 0 \tag{6}$$

By substituting equation (1) into equation (6), we have two different solutions of the polarizing wave, i.e. ordinary wave and extraordinary wave, respectively:

$$k_\perp^2 = \omega^2\mu_0\varepsilon_\perp \tag{7}$$

$$k_{\text{eff}}^2 = \frac{\omega^2\mu_0\varepsilon_\parallel\varepsilon_\perp}{\varepsilon_\parallel\sin^2\theta\cos^2\varphi + \varepsilon_\perp(1-\sin^2\theta\cos^2\varphi)} \tag{8}$$

And their corresponding refractive indices are:

$$n_o = \sqrt{\frac{\varepsilon_\perp}{\varepsilon_0}} = n_\perp \tag{9}$$

$$n_{\text{eff}} = \sqrt{\frac{\varepsilon_{\parallel}\varepsilon_{\perp}/\varepsilon_0}{\varepsilon_{\parallel}\sin^2\theta\cos^2\varphi+\varepsilon_{\perp}(1-\sin^2\theta\cos^2\varphi)}} = \frac{n_{\perp}n_{\parallel}}{\sqrt{n_{\perp}^2(1-\sin^2\theta\cos^2\varphi)+n_{\parallel}^2\sin^2\theta\cos^2\varphi}} \tag{10}$$

where $n_{\parallel} = \sqrt{\frac{\varepsilon_{\parallel}}{\varepsilon_0}}$, ε_0 is the vacuum permittivity. It can be found that the effective index of ordinary wave (*o*-wave) is constant $n_{\perp}$, whose polarization is perpendicular to the plane composed by the director and the wave vector ($\hat{\mathbf{n}} - \mathbf{k}$) as shown in Fig. S1. In contrast, the polarization of the extraordinary wave (*e*-wave) is in the ($\hat{\mathbf{n}} - \mathbf{k}$) plane, and its effective index n_{eff} depends on the intersection angle between the wavevector and the local director orientations of the NLC. When the directors are in-plane distributed (θ=90°), equation (10) is simplified as $n_{\text{eff}} = \frac{n_{\perp}n_{\parallel}}{\sqrt{n_{\perp}^2\sin^2\varphi+n_{\parallel}^2\cos^2\varphi}}$. The effective index of the *e*-wave in NLC film is shown in Fig. S2.

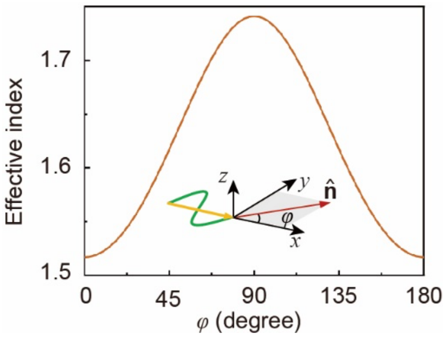

FIG. S2. Effective index of the extraordinary wave propagating in the NLC medium (E7) versus the in-plane angle φ of the director. Inset: Schematic of intersection angle between the wavevector and the director. The linear polarization of input light is along the y-direction.

In addition, considering the propagation of *o*-wave in a uniform NLC film and the fact that the NLC film thickness (10-20 μm in our platform) is much larger than the light wavelength, the effective index for *o*-wave and *e*-wave can be well approximated by the bulk-limit value of $n_{\perp}$ and n_{eff}, respectively, as shown in Fig. S3.

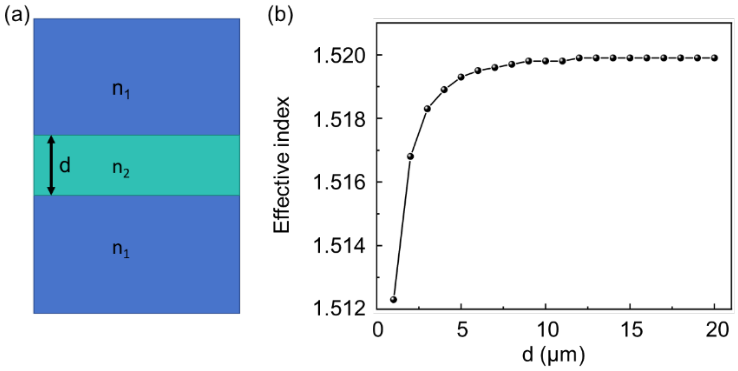

FIG. S3. (a) Schematic of planar waveguide structure for modeling light field in liquid crystal cell. (b) Calculated effective index of the fundamental mode of light depending on the thickness of liquid

crystal film. Here we consider the o-wave of liquid crystal with refractive index n_2=1.52, and the glass cladding index n_1=1.5. The cladding thickness is assumed to be infinite since the practical glass thickness is ~1.1 mm, which is far larger than the core thickness.

Let us consider the propagation of light in a two-dimensional (2D) inhomogeneous NLC film, the director of which is randomly distributed. We suppose the wavevector of light is along the *x*-direction (Fig. S1), and the input light is of a linear polarization **E**=*E* (0, cosα, sinα), α is the linear polarization angle relating to the *y*-direction. The input light field can be written as a linear combination of *o*-wave and *e*-wave:

$$\mathbf{E} = \sqrt{1-\rho}\mathbf{E_1} + \sqrt{\rho}\mathbf{E_2} \tag{11}$$

where $\mathbf{E_1}$ and $\mathbf{E_2}$ are the electric field of *o*-wave and *e*-wave, respectively; and $\rho = \frac{(\sin\theta\sin\varphi\cos\alpha+\cos\theta\sin\alpha)^2}{\cos^2\theta+\sin^2\theta\sin^2\varphi}$ (12) is the local formation factor (*e*-wave energy ratio), depending on the linear-polarization α and NLC director $\hat{\mathbf{n}}$. The governing two-dimensional (2D) Helmholtz equation for a linear-polarization light field $\mathbf{E}$ in an inhomogeneous NLC film (the director is randomly distributed) can be written in the following form given the condition that the dielectric anisotropy is slowly changed as compared to the light wavelength:

$$\nabla_\perp{}^2\mathbf{E} + \omega^2\mu_0\overleftrightarrow{\varepsilon}(x,y)\mathbf{E} = 0 \tag{13}$$

where $\nabla_\perp{}^2 = \frac{\partial^2}{\partial x^2} + \frac{\partial^2}{\partial y^2}$. By substituting equation (11) into (13), we have:

$$\nabla_\perp{}^2(\sqrt{1-\rho}\mathbf{E_1} + \sqrt{\rho}\mathbf{E_2}) + \omega^2\mu_0\overleftrightarrow{\varepsilon}(x,y)(\sqrt{1-\rho}\mathbf{E_1} + \sqrt{\rho}\mathbf{E_2}) = 0 \tag{14}$$

and based on equations (9-10) we have:

$$\nabla_\perp{}^2\mathbf{E_1} + \omega^2\mu_0\overleftrightarrow{\varepsilon}(x,y)\mathbf{E_1} = \nabla_\perp{}^2\mathbf{E_1} + k_0^2n_1^2\mathbf{E_1} = 0 \tag{15}$$

$$\nabla_\perp{}^2\mathbf{E_2} + \omega^2\mu_0\overleftrightarrow{\varepsilon}(x,y)\mathbf{E_2} = \nabla_\perp{}^2\mathbf{E_2} + k_0^2n_{\mathrm{eff}}^2(x,y)\mathbf{E_2} = 0 \tag{16}$$

To consider the branched flow of light in a generic geometry, the above equations for light propagation in the local coordinates have to be projected into the global average frame. If the director for the NLC in the region of interest is $\hat{\mathbf{n}}\langle\hat{\boldsymbol{n}}\rangle$, then this vector defines the literal ordinary and extraordinary polarizations. We decompose the total electric field into these polarizations as follows, $\mathbf{E} = \varphi\mathbf{E_o} + \psi\mathbf{E_e}$ where $\mathbf{E_o}$ and $\mathbf{E_e}$ are the normalized polarization vectors defined according to the literal ordinary and extraordinary polarizations, φ and ψ are the complex wave amplitudes for these polarizations. Projecting Eqs. (15) and (16) into these polarizations, under the paraxial approximation when the scattering is weak, we obtain the following equations,

$$\nabla_{\perp}{}^{2}\varphi + k_0^2 n_1^2 \varphi = 0 \tag{17}$$

$$\nabla_{\perp}{}^{2}\psi + k_0^2 n_{\text{eff}}^2(x,y)\psi = 0. \tag{18}$$

By adding the averaged squared effective index for the extraordinary wave,

$$\bar{n}^2 = \langle n_{\text{eff}}^2(x,y)\rangle \tag{19}$$

we obtain the following equation for the *e*-wave,

$$-\nabla_{\perp}{}^{2}\psi + k_0^2(\bar{n}^2 - n_{\text{eff}}^2(x,y))\psi = k_0^2\bar{n}^2\psi \tag{20}$$

Equation (20) resembles the time-independent Schrödinger equation where the total energy E_{tot} is $k_0^2\bar{n}^2$ and the optical potential energy is $V(\mathbf{r}) = k_0{}^2(\bar{n}^2 - n_{\text{eff}}^2(x,y))$. The latter serves as the weak disorder potential for the *e*-wave with a zero average value $\langle V(\mathbf{r})\rangle = 0$, which is the cause of the emergence of the branched flow of light. Consequently, the distribution of the NLC director $\hat{\mathbf{n}}(x,y)$ (Fig. S4a) can be mapped to the profile of the effective index (Fig. S4b). The optical potential energy $V(\mathbf{r})$ is then obtained and shown in Fig. S4c.

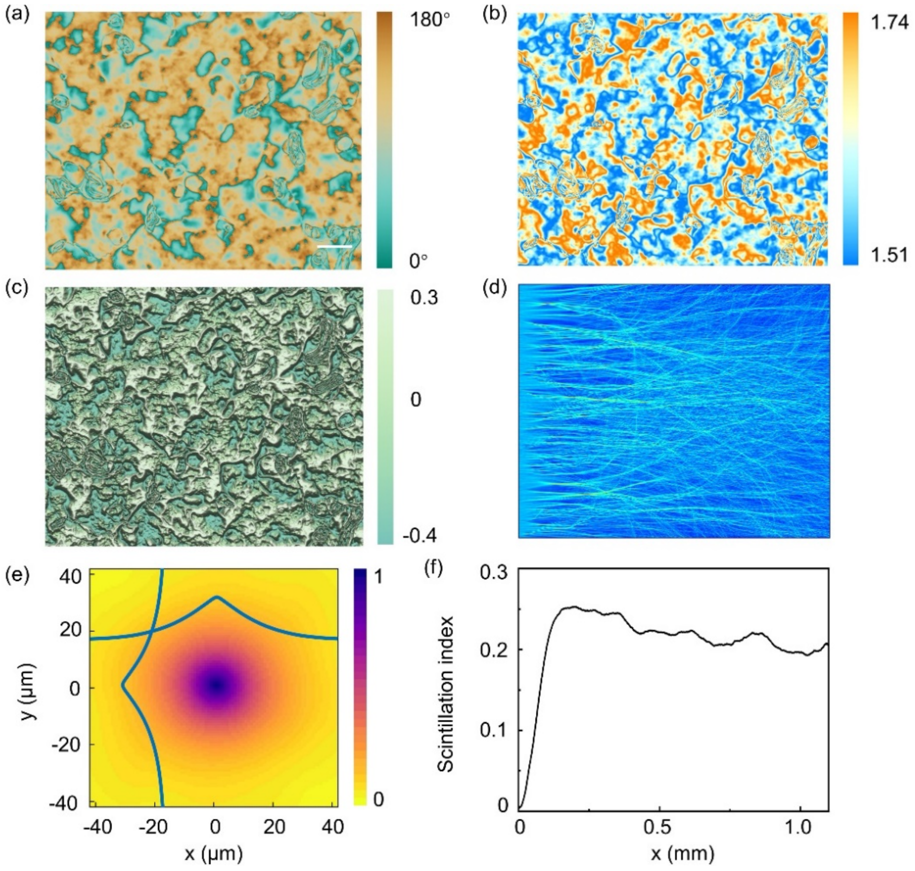

FIG. S4. Construction of equivalent potential by inhomogeneous dielectric anisotropy of nematic liquid crystal (NLC) and simulations of branched flow of light. (a) Typical random director distribution of a NLC film measured by a polarization interference microscopy. (b) Reconstructed

effective index distribution for e-wave based on the director distributions from (a) based on equation (10). (c) Equivalent optical potential landscape, for the propagating e-wave inside the NLC film. (d) Numerical simulation of plane-wave input in the effective index landscape extracted from (b), and its evolution clearly shows the branched feature. The light wavelength is set as 532 nm. The scale bar is 100 μm. (e) Autocorrelation function of the equivalent optical potential extracted from (c). The blue line represents the horizontal and vertical cross-sectional output. (f) Average scintillation index of the four simulation results is taken as a function of the propagation distance.

Theoretically, the branched flow is mainly determined by two parameters of disordered potential[1]: the strength of the potential ϵ and the correlation length l_c. In two-dimensional systems, the correlation function of the optical potential can be written as $c(\mathbf{r}) = \langle V(\mathbf{r}' + \mathbf{r})V(\mathbf{r}')\rangle = 4\epsilon^2 E_{tot}^2 f(\mathbf{r}\cdot\mathbf{r}/l_c^2)$ with $\epsilon = \sqrt{\langle V^2\rangle}/2E_{\mathrm{tot}}$ and $f(0) = 1$. Note that for branched flows, the formation of branches does not depend on the specific structure of the disorder potential, or even on the specific form of the correlation function. For instance, the correlation function $c(\mathbf{r})$ can be Gaussian or exponential attenuation or any other smooth function. The typical correlation function of anisotropic NLC film is shown in Fig. S4e, and the extracted correlation length is approximately 12 μm. It can be seen that the optical potential energy induced by the inhomogeneous anisotropic NLC is weak and disordered since the potential strength is small, i.e., $\epsilon = 0.5\sqrt{\frac{\langle n_{\mathrm{eff}}^4\rangle}{\bar{n}^4} - 1} \approx 0.046$. Therefore, the propagation of light is dominated by small-angle scatterings. This regime of weak disorder potentials also justifies the paraxial approximation. In other words, the overall effect of the inter-polarization scattering is negligible after averaging over the whole region of light propagation. The main effect of scattering is to cause branched flow in the extraordinary waves. Here, the branches are generated by caustics appearing in the optical flow when the light ray starts to cross. The distance d_f from the starting point to the first concentrated caustics position satisfies a statistical length scale[2,3]:

$$d_f \propto l_c \epsilon^{-2/3} \tag{21}$$

This first branching distance is a key parameter in branched flows. The proportionality factor depends on the mathematical form of the correlation function[3]. And it is found that the scaling of d_f in equation (21) will break down when the potential strength $\epsilon > 0.1$. Thus in our experimental setup ($\epsilon \approx 0.046$), the scaling law of first branching is effective.

As shown in Fig. S4d, the branched flow of light is featured with high caustics density and high intensity in the propagating light waves from the numerical simulations. The conventional method of extracting the first branching distance is through tracing the scintillation index as a function of the propagation distance[3]:

$$S(x) = \frac{\langle I(x,y)^2 \rangle}{\langle I(x,y) \rangle^2} - 1 \tag{22}$$

where $I\,(x,y)$ is the local optical intensity and the average is taken over the transverse y-coordinate and over different ensembles of the same device. We simulate the evolving optical fields at several realizations and calculate the average scintillation index curve as shown in Fig. S4f. The prominent peak of the scintillation curve indicates the first branch distance. Figure S5 shows the experimental measurements of branched fields and the scintillation index calculations. Note that the scintillation index curve is averaged over several measurements. We also numerically study the dielectric loss of NLC on the branched flow fields (Fig. S6). It can be found that the dielectric loss significantly reduces the intensity of propagating fields and the branched features can only be observed around the first branched distance, which agrees with our experimental results as discussed later.

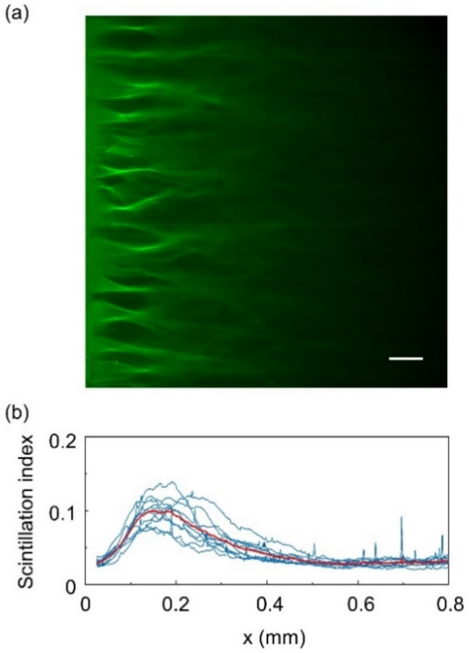

FIG. S5. Scintillation index of branched flow of light. (a) Distribution of branched light field with a quasi-plane-wave input. The scale bar is 100 μm. (b) Scintillation index of 10 measurements, realized by measuring the branched flow profiles in different sub-region of a same NLC cell. The red line is the average experimental scintillation index. The average result is shown in Fig. 3c in the main text.

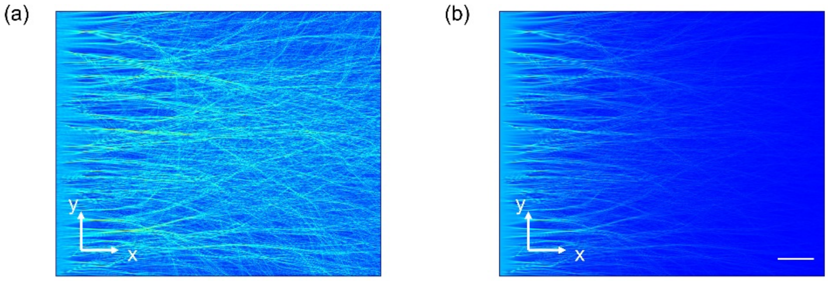

FIG. S6. The dielectric loss of NLC influence on the profiles of branched flow of light. Numerical simulation results of branched flow of light polarized along the *y*-direction (a) without loss and (b) with loss. In the simulations, the boundary condition is set as scattering boundary conditions. The imaginary part of refractive index is set as 10^{-3}, and the light wavelength is set as 532 nm. The scale bar is 100 μm.

Supplementary Note 2: Experimental setup and optical characterizations

The schlieren textures of NLC film can be directly observed under a polarized microscope as shown in Fig. S7. The important part of a polarized microscope is the polarizing components, i.e., the polarizer and the analyzer. Polarization directions of the polarizer in front of the sample and the analyzer behind the sample are perpendicular to each other. When there is no detected object or it is isotropic, the field of view is dark. If the detected object has birefringence, the brightness of the field of view is determined by the direction of the optical axis. We can find that with the increase of gating voltages, the NLC molecules are gradually aligned with electric field since the inhomogeneous brightness of the images is considerably decreased. Intriguingly, the topological defects are maintained and visible despite the gating voltages. The repeatability of director distributions with gating voltages guarantees the reversibly electrical control of branched flow of light in NLC film.

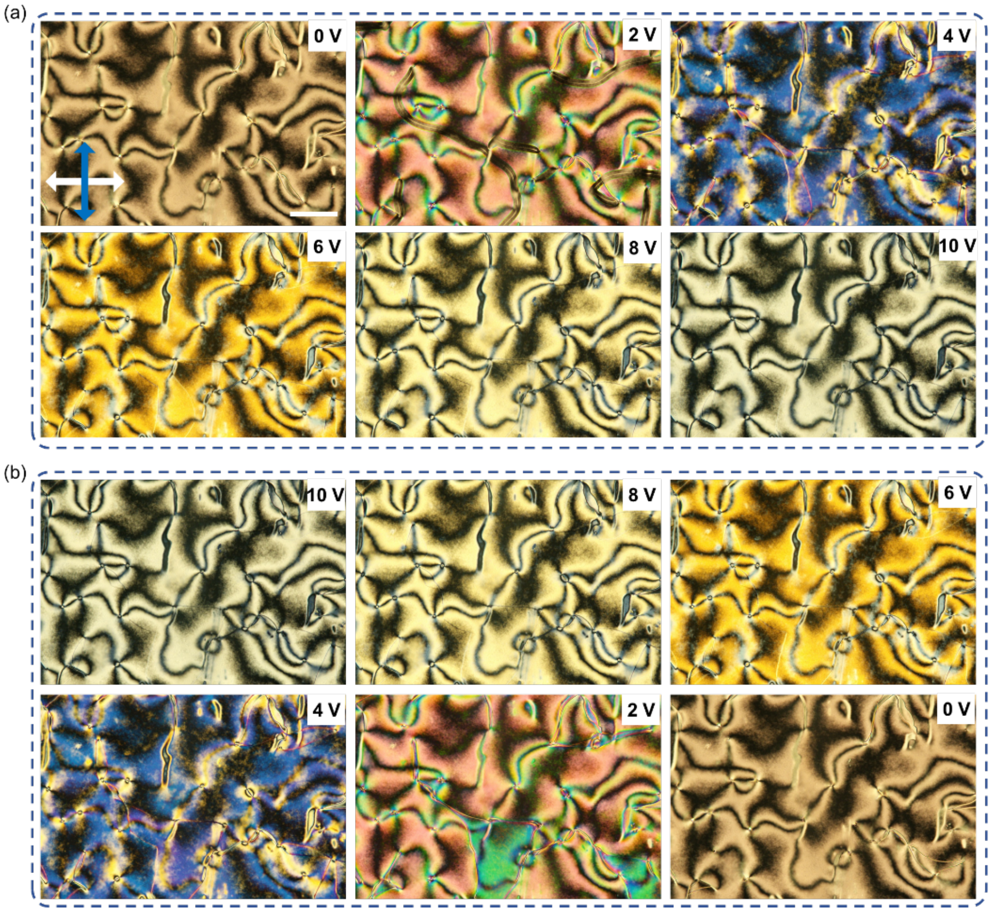

FIG. S7. (a, b) Polarized microscope image of disordered NLC film (a) with the increase of gating voltages and (b) with the decrease of gating voltages. The scale bar is 100 μm. The arrows indicate the polarizer and analyzer directions.

With the increase of the voltage bias along the *z*-direction (i.e., perpendicular to the film plane), the NCL molecules are gradually aligned towards the *z*-direction. Such a change in the molecules' orientation suppresses the schlieren textures. Indeed, in Fig. S7a, the schlieren textures gradually become blurry as the voltage bias increases. Because the voltage bias is not very large, and the surface forces and torques pin the topological defects (mainly disclinations), thus these topological defects survive the external voltage biases and can be revived when the voltage bias is tuned down, as shown in Fig. S7b. This remarkable property makes the electrical control of the branched flow of light reversible.

In order to generate and observe branched flow of light, we have built two experimental configurations as shown in Fig. S8, to couple the focused Gaussian beam or quasi-plane-wave into the NLC film. Note that the intrinsic strong light scattering in the NLC medium from the orientation fluctuations enables direct observations of evolving light fields, imaged by a microscope system,

without any fluorescent molecules doping. As schematically presented in Fig. S8a, the laser (532 nm, Changchun Laser Optoelectronics Technology Co., Ltd, MW-SL-532/100mW) passed through a neutral density filter allowing for adjusting the beam power, and the polarization is aligned along *y*-direction by a linear polarizer. The linear polarization direction of the light beam can be adjusted by a half-wave plate. The Olympus objective with 10× magnification and a numerical aperture of 0.25 shaped the collimated beam into a focused Gaussian beam. As for the quasi-plane-wave input setup, the linearly polarized beam is expanded by a concave lens (f=50 mm) and then passes through a convex cylindrical lens (f=100 mm) to generate a broad elliptic beam to approximate a plane-wave beam (Fig. S8b).

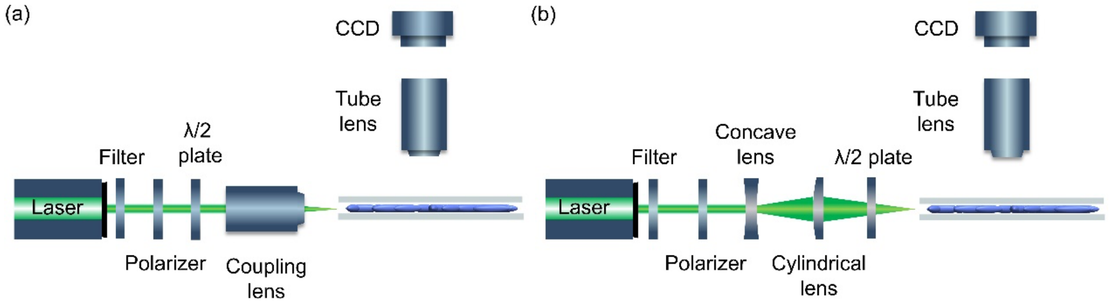

FIG. S8. Schematic diagram of the optical setup for coupling (a) a Gaussian beam and (b) a wide elliptical beam into the planar NLC device.

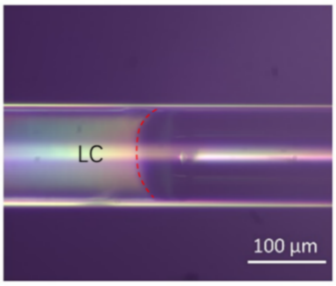

FIG. S9. Microscope image of liquid crystal infiltrated in a silica glass tube. The red dashed line labels the curved air-liquid-crystal-glass interface.

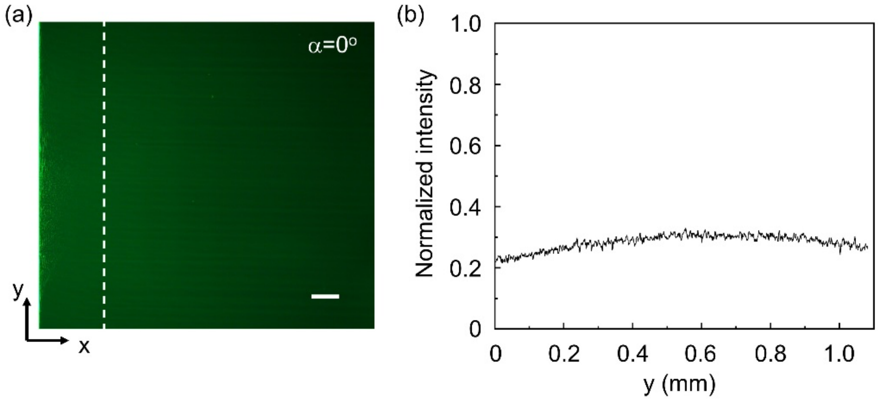

FIG. S10. (a) The light field in a homogeneous unperturbed cell with director along y coupled by a quasi-plane-wave with polarization along y-direction. (b) The cross-section intensity distribution at the dashed line position of (a). The scale bar is 100 μm.

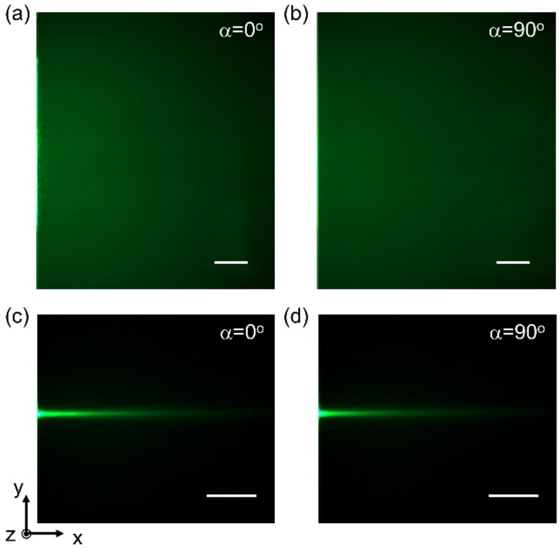

FIG. S11. Optical field landscapes in a uniform NLC cell for quasi-plane-wave input of light polarization (a) along y-direction, and (b) along z-direction. Propagating field landscapes in a uniform NLC cell for Gaussian beam input with input polarization (c) along y-direction, (d) z-direction. The scale bar is 200 μm.

To study the curved air/NLC interface, we infiltrate NLC into a silica glass tube as shown in Fig. S9. The contact angle of ~37° between glass and NLC is determined by their surface tension coefficient and interface interaction[4]. The cured air/NLC interface can influence the light coupling. We also fabricate a uniform alignment NLC cell and measure the landscapes of propagating fields as benchmark. Figure S10 shows the propagating fields in a uniform cell with quasi-plane-wave input. These nearly uniform fields indicate that the curved injection interface has a negligible effect

on the propagating fields. We also characterize the influence of polarization of light on the propagating fields in the uniform cell. Neither branched patterns nor caustics can be observed for both the quasi-plane-wave input and Gaussian beam input, as shown in Fig. S11. In addition, the propagating fields show similar landscapes without branching structures for the light polarization along the in-plane (y-direction) and out-of-plane (z-direction). These experimental results unambiguously clarify the effects of inhomogeneous director distributions for branched flow generation.

Supplementary Note 3: Tunable branched flow of light in nematic liquid crystal

Figure S12 shows the experimental results for liquid crystal cell of thickness 10 μm and 20 μm, respectively, and they exhibit similar branched flow features under different gating voltages, which help clarify that waveguiding effect can be negligible in our platform.

We measure the landscapes of propagating fields in a disordered NLC film with gradual change of the gating voltages, as shown in Fig. S13. This electrical voltage has two main effects: First, the applied electric voltages suppress the fluctuations of the potential energy; Second, the alignment of optical axes of NLC induces excitations of the ordinary wave, which does not experience inhomogeneous potential. In addition, when the gating voltages are larger than 5 V, the surface anchoring molecules will also be aligned and the NLC defects are significantly reduced. Consequently, the optical field landscape shows much smooth profiles. Note that the light scattering from NLC is also reduced compared with that of low-gating voltages since the electric field suppresses the director orientation fluctuations.

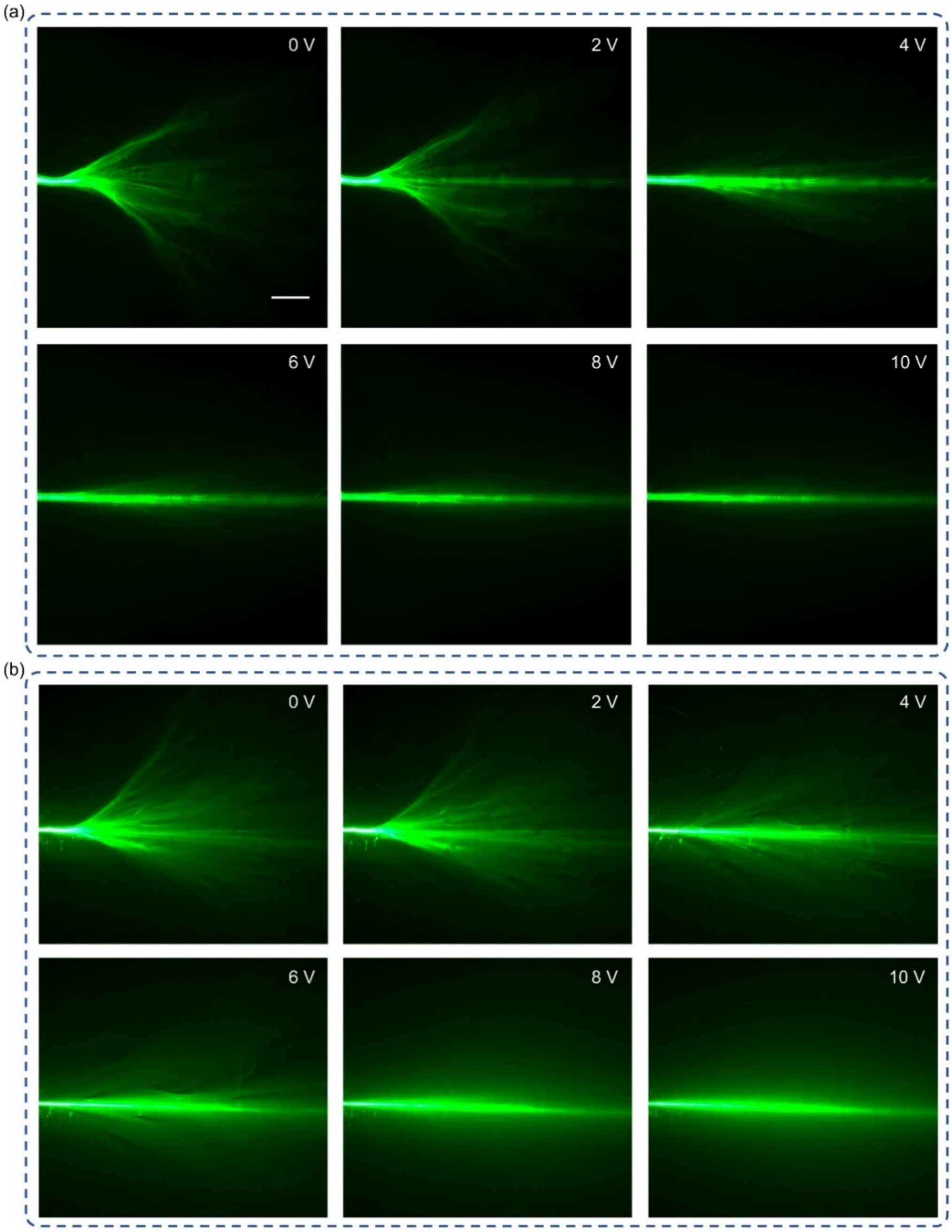

FIG.S12. Electrical control of branched flow of light in disordered NLC medium of different film thickness: (a) NLC film thickness of 10 μm, (b) NLC film thickness of 20 μm. The scale bar is 100 μm. The branched flow profiles in different-thickness NLC film follow a similar rule, for example, the first branched distance, and their branched pattern can be switched on/off by the electric voltages.

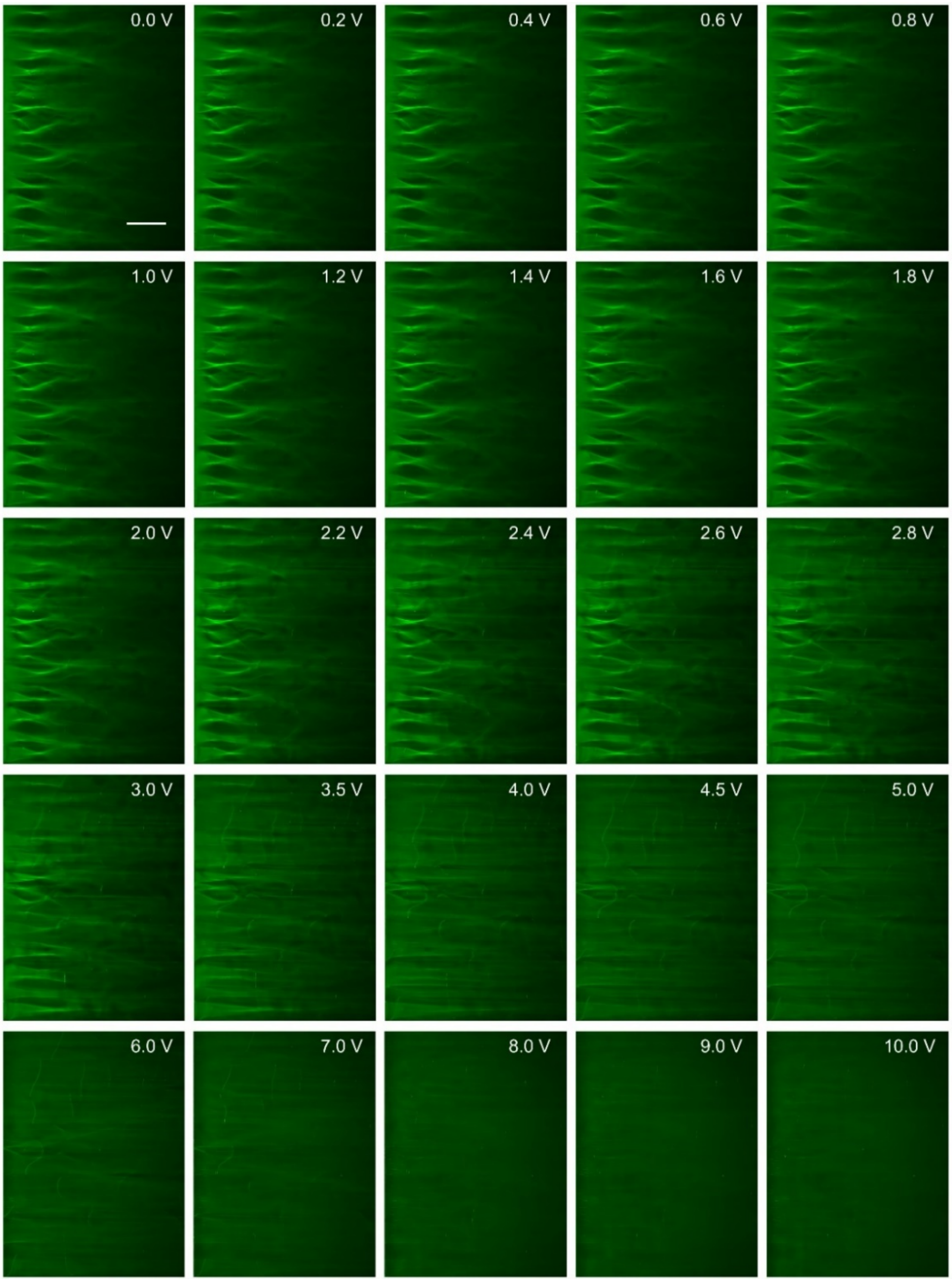

FIG. S13. Electric control of branched flow of light with plane-wave input. The scale bar is 200 μm. With the increase of applied voltages, the concentrated caustics gradually disappear due to the field-alignment of NLC molecules.

Under the condition of quasi-plane-wave coupling, we calculate the probability distribution of light intensity upon 0 V and 10 V as shown in Fig. S14. These statistical characteristics are similar to those of Gaussian beam coupling (Fig. 2b-c in the main text).

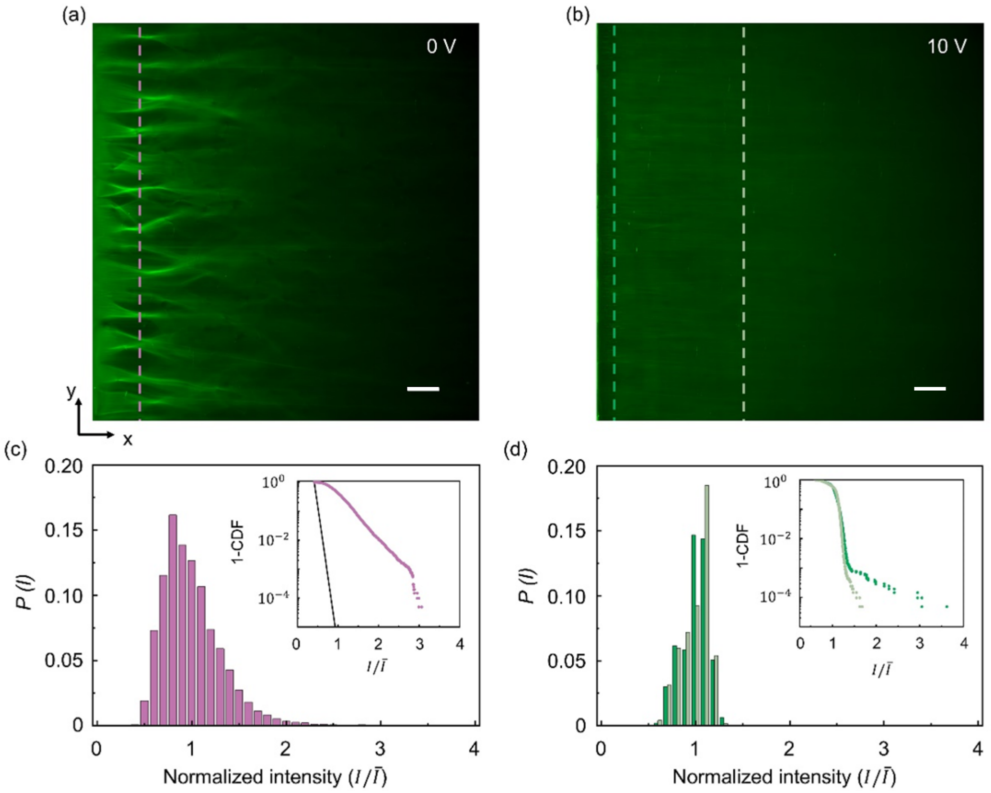

FIG. S14. Measured profiles of the optical field with quasi-plane-wave incident for different electric voltages: (a) 0 V and (b) 10 V. The scale bar is 100 μm. (c) Statistical distribution of the cross-section intensity at the dashed line in (a). The inset shows the cumulative distribution function (CDF) of the cross-section intensity in semi-log axis. The black curve represents the CDF of Rayleigh distribution. (d) Probability distributions of the optical intensity with an electric voltage bias of 10 V at the incident (olive green columns) and at the white-dashed line in (b) (greyish green columns). Inset: CDF of the two distributions of the optical intensity.

The Fréedericksz transition is field-induced reorientation of the liquid crystal. The Fréedericksz transition and the threshold voltage V_c can be calculated via the following formula[5]:

$$V_c = \pi\sqrt{\frac{K_{11}}{\varepsilon_0 \Delta\varepsilon}} \tag{23}$$

where splay elastic constant $K_{11} = 11.09$ pN, ε_0 is vacuum dielectric constant, and the dielectric anisotropy $\Delta\varepsilon = \varepsilon_{\parallel} - \varepsilon_{\perp} = 13.9$ can be obtained for E7 material[6]. Thus the calculated Fréedericksz threshold voltage is ~1.8 V_{pp}, which is close to the experimental results (~1.6 V_{pp}).

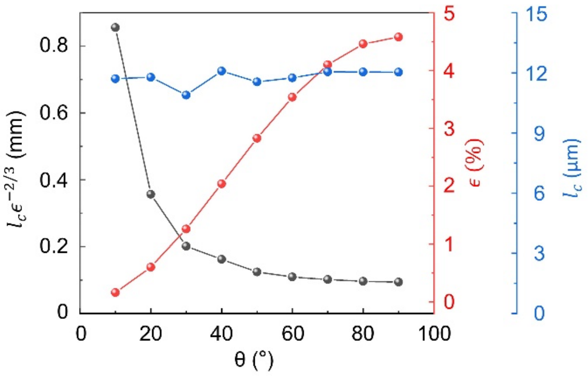

FIG. S15. The first branched position $l_c\epsilon^{-2/3}$, potential strength ϵ and correlation length l_c change with the polar orientation angle of NLC director. The theoretical calculations show that the change of first branched position is mainly contributed by the change potential strength due to the globally orientations of NLC director. For experiments, the orientations of NLC director are manipulated by gating electric voltages.

The electro-optical response of NLC medium enables that the director $\hat{\mathbf{n}}(\mathbf{r})$ can be aligned with the gating electric field. We suppose that the director is initially in-plane oriented (θ=90°), and the probe light polarization along y-direction according to the experimental setup. When the polar angle θ of NLC director collectively decreases from 90° to 0° driven by electric voltage, the effective optical potential can be modulated, thus the branched flow of light is dynamically manipulated. First, we calculate the curve of the first branching position $l_c\epsilon^{-2/3}$ vs polar angle θ as shown in Fig. S15. Here the initial optical potential is obtained from the experiment measurements. Theoretically, the first branching position only slightly increases when θ is in the range of 50°-90°. For the experiment, the position of the first branching is conventionally extracted by the peak of the average scintillation index from multiple realizations. When the out-of-plane polar angle is large (θ=50°-90°), theoretical predictions show that the first branching is not sensitive to the polar angle, thus it should be difficult to directly resolve the position shift of first branching in this stage, which agrees qualitatively with experimental observations. While, when the polar angle is smaller than 50°, the excitation of o-wave resulting from the birefringence effect is dominant, which will significantly mix with the e-wave fields. The detailed discussions on this issue are as follows.

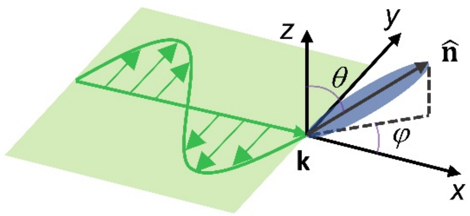

FIG. S16. Schematic of NLC molecules interactions with polarized light incidence. The wave-vector **k** is along *x* direction, and the polarization is along *y*-direction.

Light field with initial polarization along *y*-direction (Fig. S16) can be decomposed into *o*-wave and *e*-wave for an out-of-plane alignment NLC. Theoretically, only *e*-wave in planar NLC film experiences non-uniform equivalent refractive index and forms branch flow. In Section 1, we define the local formation factor $\rho = \frac{(\sin\theta\sin\varphi\cos\alpha+\cos\theta\sin\alpha)^2}{\cos^2\theta+\sin^2\theta\sin^2\varphi}$ to quantitatively characterize the *e*-wave excitation ratio. In particular, when $\alpha = 0°$, i.e., light polarization along *y*-direction, $\rho = \frac{(\sin\theta\sin\varphi)^2}{\cos^2\theta+\sin^2\theta\sin^2\varphi}$. We take the average formation factor $\langle\rho\rangle = \bar{\rho}$ of the entire observation area to characterize the global excitation ratio of *e*-wave. Suppose that *o*-wave only provides uniform background field intensity, and the scintillation on formation factor $\bar{\rho}$ can be derived:

$$S = \frac{\langle[I\bar{\rho}+\langle I(1-\bar{\rho})\rangle]^2\rangle-\langle I\bar{\rho}+\langle I(1-\bar{\rho})\rangle\rangle^2}{\langle I\bar{\rho}+\langle I(1-\bar{\rho})\rangle\rangle^2} = \bar{\rho}^2 S_0 \tag{24}$$

where S and S_0 correspond to the cases with and without *o*-wave excitations, respectively. In experimental measurements, we define the scintillation contrast as $SC=S_{\mathrm{peak}}-S_{\mathrm{base}}$ as a typical feature of the scintillation curve. Thereafter, equation (24) can also be approximately written as

$$SC = SC_0\bar{\rho}^2 \tag{25}$$

Since $\bar{\rho}$ depends on the gating voltages on NLC film, the SC can be dynamically controlled as shown in Fig. 3d in the main text.

To correlate the deflection of the polar angle to the electric voltage, we simulate the NLC orientations in commercial software Tech Wiz-LCD-3D. The polar angle of the NLC director under various voltages is calculated as shown in Fig. S17. Without loss of generality, we set the NLC molecules initially in-plane aligned without applied electric field (Fig. S17a). The ITO driving electrode is coated on the bottom glass substrate to produce a driving field. Figure S17b shows the cross-sectional view of the arrangement of NLC molecules in a planar cell when the driving voltage

(V_{pp}) is 5 V. From the deflection angle curve with gating voltages, the NLC molecules respond sensitively with voltages in the middle-cell position; on the contrast, due to the strong anchoring effect of glass, the NLC molecules at the surface layer (~ 2 μm) shows much smaller deflection angle than that of the middle-cell position under a same driven voltage as shown in Fig. S17c. We note that the surface layer NLC contributed to the experimental observations, since the observed branched flow profiles only considerably changed with gating voltages larger than 2V as shown in Fig. S13.

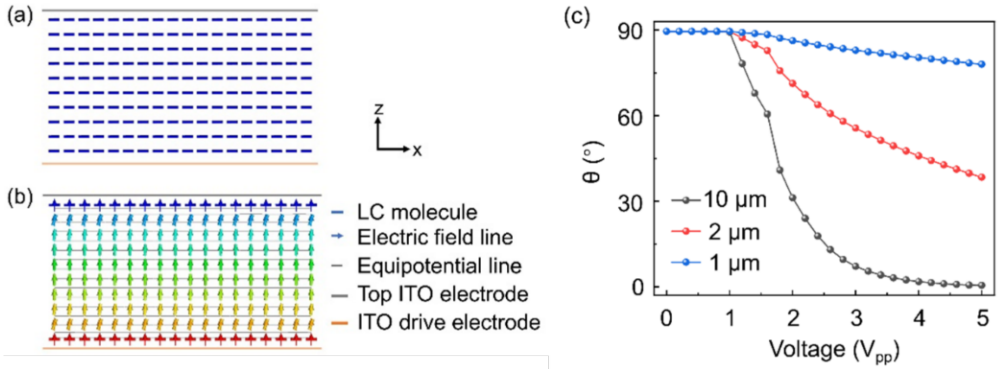

FIG. S17. Cross-sectional view of NLC director when the driving voltage (V_{pp}) is (a) 0 V and (b) 5 V. (c) The polar angle of NLC molecules at the positions of 1 μm, 2 μm and 10 μm from the driving electrode changes with the voltage. The simulation parameters are as following: cell gap is 20 μm, the anisotropy of permittivity of NLC is $\Delta\varepsilon = 13.9$.

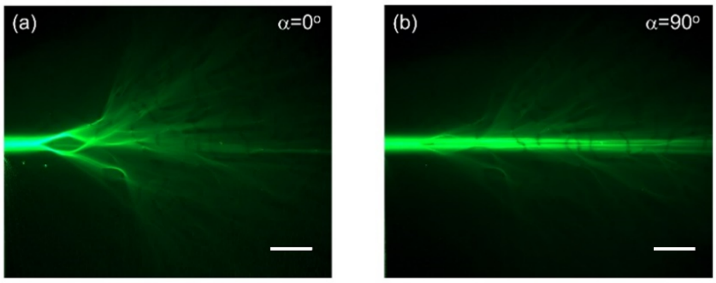

FIG. S18. Optical field landscapes in disordered liquid crystal cell for (a) *e*-wave excitation (in-plane polarization), (b) *o*-wave excitation (out-of-plane polarization). The scale bar is 200 μm.

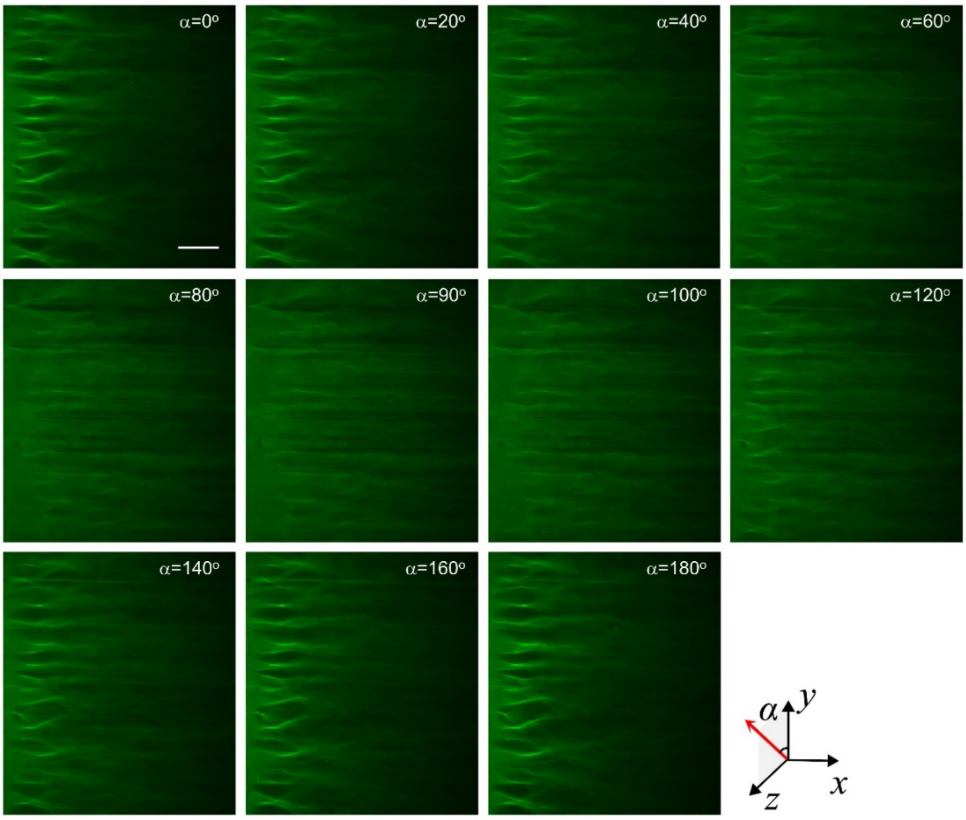

FIG. S19. Observations of polarization-dependent branched flow of light with quasi-plane-wave input. The scale bar is 200 μm.

The branched flows can also be manipulated by the polarization of the incident light due to the anisotropic optical properties of the liquid crystal and the planar film setup. We first measure the optical landscapes in a disordered liquid crystal cell for different linear polarizations of the Gaussian beam, as shown in Fig. S18. The e-wave excited fields manifest typical branched-flow geometry, on the contrary, the o-wave excited fields show natural diffraction features with increasing width and decreasing on-axis intensity. Note that there exist minor branched structures even for o-wave excitations due to the small pretilt angle of liquid crystals in the interface of the glass substrate. As for the quasi-plane-wave input, the experimental results are shown in Fig. S19. It is found that when the input light polarization angle is tuned from 0° to 90°, the branched flow with concentrated caustics is gradually reduced, which unambiguously certifies that only extraordinary wave contributes to the branched flow of light phenomenon. Theoretically, by simply changing the polarization angle (α) of the input quasi-plane-wave under the gating free condition, the proportion of e-waves that forms branched flow of light can be tuned. From equation (12), when NLC molecules are in-plane distributed, i.e., $\theta = 90^{\circ}$, $\rho = \cos^2\alpha$, which is independent on the in-plane

azimuthal angle. From equation (25), we obtain a simple formula $SC=\cos^4\alpha$ for statistical features of branched flow under different polarized light input. The experimental results fit well with theoretical predictions as shown in Fig. 4e in the main text.

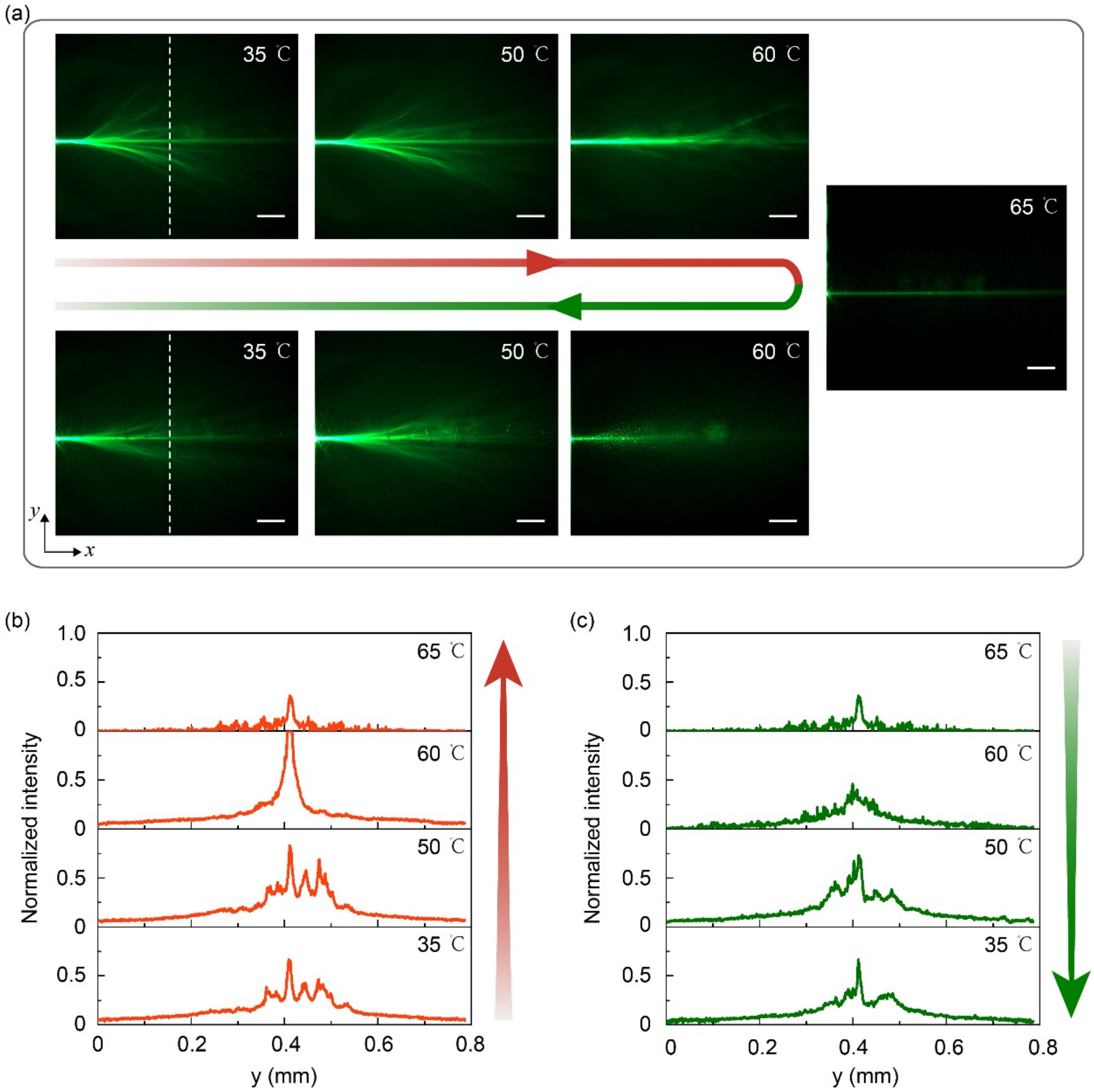

FIG. S20. Temperature tuning of the branched flow of light. (a) Thermal switch off and on of the branched flow of light upon temperature cycling. The scale bar is 100 μm. (b-c) The single-shot traces of cross-section optical intensity at the white-dashed line in (a) during the processes of heating (b) and cooling (c).

Indeed, thermal tuning is another useful technique for the manipulation of photons in liquid crystals. We agree with the reviewer that using temperature control can also tune the branched flow of light as shown in Fig. S20. Nevertheless, we find that the thermal tuning method is highly irreversible by comparing the cross-section field distribution of Fig. S20b and Fig. S20c. It is because that the temperature cycling can change the mesoscopic structures (i.e., the director

distribution) of the liquid crystal probably due to the collision of the growing isotropic-nematic interface during the cooling process, which can result in a higher energy metastable state. In contrast, electrical tuning method can realize highly reversible manipulations of the branched patterns since the topological structures of liquid crystals are stable under such tuning (if the electrical field is not too high).

Supplementary Note 4: Statistics of branch density

When a plane-wave light field passes through a weak correlated disorder potential, it leads to a pronounced branching of the flow with concentrated caustics. The branched density, defined as caustic numbers per unit length on average at a certain distance from the source, is an important statistical feature of branched flow. To extract the branched quantity, we calculate the branched number as half of the caustic peaks, as shown in Fig. S21. Recently, Metzger et al.,[7] deduced an analytical expression of branched density to study the statistics of branching in two-dimensional Hamiltonian systems. Here we briefly discuss the theory as follows.

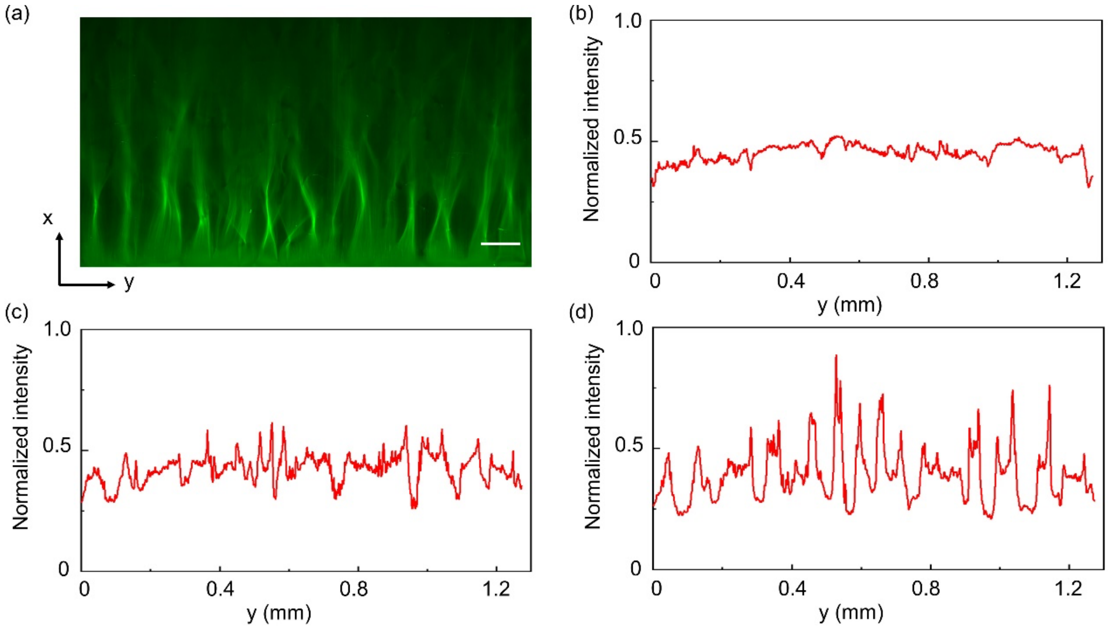

FIG. S21. (a) Branched flow of light with plan-wave input. The scale bar is 200 μm. Cross-sectional intensity profile along y-direction at different propagation distances: (b) 0.02 mm, (c) 0.06 mm and (d) 0.1 mm.

When the wave propagates for a distance of several correlation lengths in the disorder potential, the cumulative deflection of the wave by the random potential will produce caustics. Two caustics form a branch, so the number of branches is half of the caustics number[7, 8]:

$$N(x) = \frac{1}{2}\lim_{L\to\infty}\frac{1}{L}\langle\int_0^L dy\,\frac{\delta(x-x_c)}{|\dot{m}(x)|}\left|\partial_y m(x)\right|\rangle \tag{26}$$

where $m(x)$ is a function that is zero when it encounters caustics, and the distance required to encounter caustics is x_c and $h(x) = \partial_y m(x)$. According to the equation (26), the probability of caustics along the trajectory can be obtained $P_c(x) = \langle\delta(x - x_c)\rangle$. In order to obtain the branch density, it is necessary to analyze the statistical characteristics of $m(x)$ and $h(x) = \partial_{y_0} m(x)$. According to Fokker-Plank equation, we can obtain the following analytical expressions in the case of quasi two-dimensional [7]:

$$\langle m^2(x)\rangle = \frac{1}{3}\left[e^{2\kappa x} + 2e^{-\kappa x}\cos(\sqrt{3}\kappa x)\right] \tag{27}$$

$$\langle h^2(x)\rangle = \frac{\sigma_2^2}{210\sigma_1^2}\left\{98e^{-\kappa x}\cos\left(\sqrt{3}\kappa x\right) + 49e^{2\kappa x} + e^{21^{1/3}2\kappa x} + 2e^{-21^{1/3}\kappa x}\cos\left[\left(7\times 3^{5/2}\right)^{1/3}\kappa x\right] - 150\right\} \tag{28}$$

where $\kappa = (\sigma_1/\sqrt{2})^{2/3}$ and $\sigma_1^2 = \frac{1}{2}\int_{-\infty}^{\infty} dx \left.\frac{\partial^4 c(x,y)}{\partial y^4}\right|_{y=0}$, $\sigma_2^2 = -\frac{1}{2}\int_{-\infty}^{\infty} dx \left.\frac{\partial^6 c(x,y)}{\partial y^6}\right|_{y=0}$. σ_1 and σ_2 can be determined by different correlation equations. Here all of the calculations are performed using Gaussian correlation disorder potential, that is, $c(\mathbf{r}) = \epsilon^2\exp(-r^2/l_c^2)$, with $\epsilon = 4.58\%$ and $l_c = 12.113\ \mu\text{m}$. Next, the expression of the probability of reaching caustics along the trajectory $P_c(x)$ needs to be derived. The complete expression has been obtained in the previous literature[7]:

$$P_c(\mathrm{x}) = \begin{cases} \left[\beta^2(4\pi\sigma_1^2)^{-1/2}x^{-5/2} + C_F(2\sigma_1^2)^{1/3}\right]e^{-\lambda_1(2\sigma_1^2)^{1/3}x - \beta^4/(12\sigma_1^2 x^3)} & if\ x \le x_1 \\ 1/x_0 & if\ x > x_1 \end{cases} \tag{29}$$

Where $\beta \approx 1.754$, $\lambda_1 \approx 0.281$, $C_F \approx 0.314$; x_0 is the average distance between two branches in the propagation direction, x_1 is the cutoff point at which the short-term drops below the long-term. Equation (29) can be further derived for short- and long-time approximations, respectively. For short term, $\langle|\dot{m}(x)|\rangle \propto 1/x_0$ at the caustics, and $\langle|h(x)|\rangle \sim \sqrt{\langle h^2(x)\rangle}$. For long-term asymptotic, $P_c \approx const$, $\dot{m}$ and h are assumed to be statistically independent, and the average absolute value increases exponentially with an exponential factor of $\lambda \approx 2.57$. Therefore, the branch density can be simplified as

$$N(\mathrm{x}) = \begin{cases} N^{short}(x) \approx c_1 x_0 P_c(x)\sqrt{\langle h^2(x)\rangle} & if\ N^{short} \le N^{long} \\ N^{long}(x) \approx c_2(\sigma_2/\sigma_1)x_0 P_c(x) e^{\lambda(x/x_0)} & if\ N^{short} > N^{long} \end{cases} \tag{28}$$

where $c_1 = 0.0061$ and $c_2 = 0.0074$. We find $x_1 \approx 0.161$ mm for dividing the short- and long-term approximation. In the short-term, the analytical solution of equation (28) agrees well with the experimental results as shown in Fig. S22. Due to the high intrinsic loss of liquid crystal medium, the intensity of light field decays fast beyond the first branch distance, thus it is difficult to resolve the long-term statistics of branch density in the current platform.

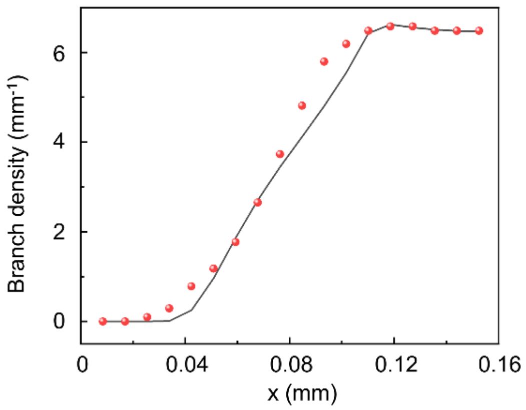

FIG. S22. Statistics of branch density with experimental results (red dots) and theoretical fittings (black curve) at short-term distance range.

Supplementary References

1. Patsyk, A., Sivan, U., Segev, M. & Bandres, M. A. Observation of branched flow of light. *Nature* **583**, 60-65 (2020).

2. Degueldre, H., Metzger, J. J., Schultheis, E. & Fleischmann, R. Channeling of branched flow in weakly scattering anisotropic media. *Phys. Rev. Lett.* **118**, 024301 (2017).

3. Barkhofen, S., Metzger, J. J., Fleischmann, R., Kuhl, U. & Stöckmann, H. J. Experimental observation of a fundamental length scale of waves in random media. *Phys. Rev. Lett.* **111**, 183902 (2013).

4. Cognard, J. The anisotropy of the surface tension of polar liquids: the case of liquid crystals. *J. Adhes.* **17**, 123-134 (1984).

5. Yang, D. K. & Wu, S. T. Fundamentals of liquid crystal devices. John Wiley & Sons, 2014.

6. Ye, W., Li, Z., Yuan, R., Zhang, P., Sun, T., Cai, M., Wang, X., Zhu, J., Sun, Y. & Xing, H. Accurate measurement of the twist elastic constant of liquid crystal by using capacitance method. *Liquid Crystals*, **46**, 349-355 (2019).

7. Metzger, J. J., Fleischmann, R. & Geisel, T. Universal statistics of branched flows. *Phys. Rev. Lett.* **105**, 020601 (2010).

8. Berry, M. V. & Upstill, C. IV. Catastrophe optics: morphologies of caustics and their diffraction patterns. *Progress in Optics* **18**, 257-346 (1980).
