# Supplementary figures and images for: Electrical tuning of branched flow of light
Source: Nat Commun. 2024 Jan 3;15:197. doi: 10.1038/s41467-023-44500-8 (PMC10764866; doi:10.1038/s41467-023-44500-8)

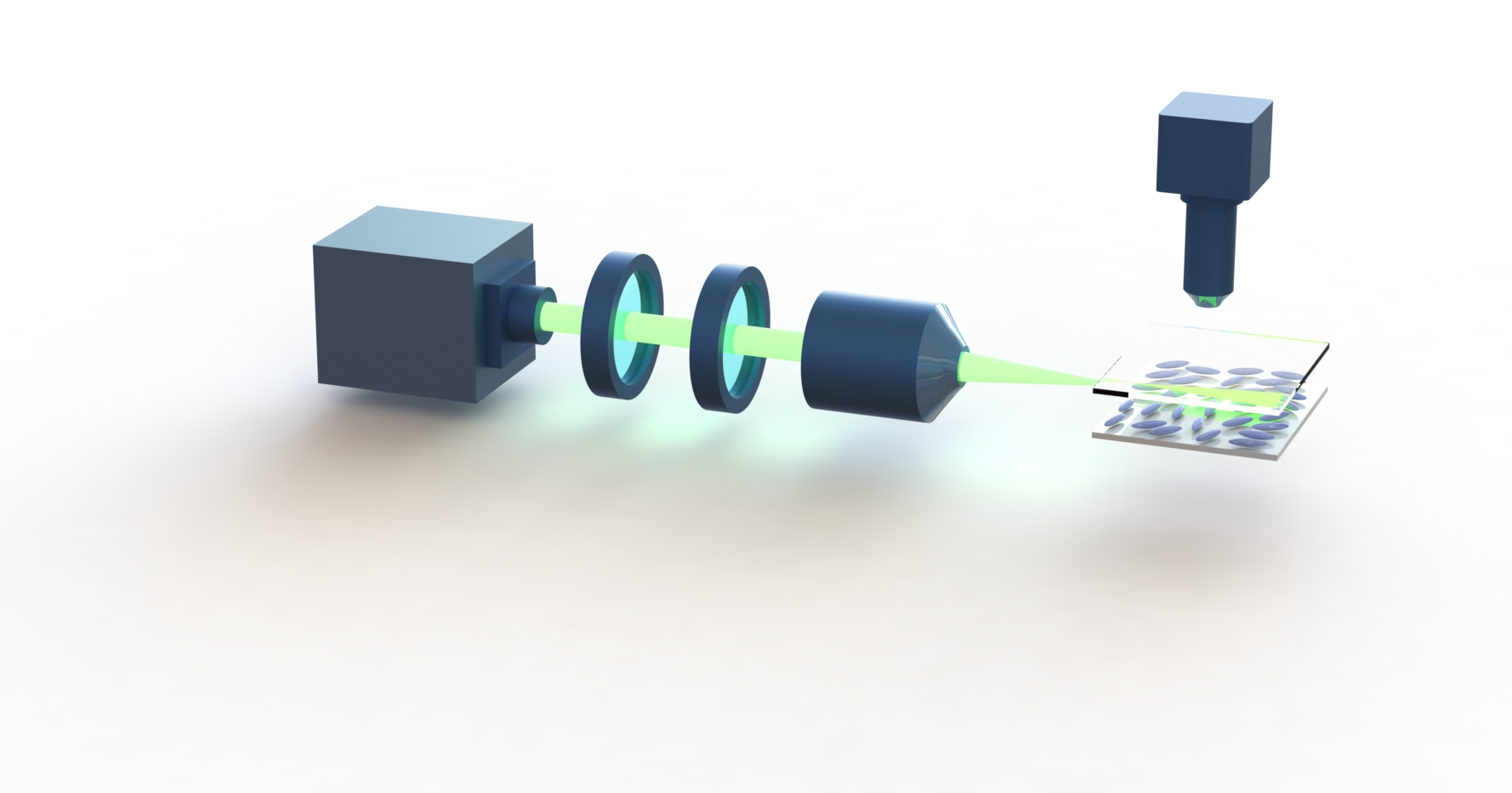

Supplement: Supplementary file 3 — Source Data [file 41467_2023_44500_MOESM3_ESM.zip › Fig1a/solidworks of fig1a/preview.JPG]
